# Effect of Valproate Monotherapy on Thyroid Function Tests and Magnesium Levels in Children With Epilepsy

**DOI:** 10.7759/cureus.39712

**Published:** 2023-05-30

**Authors:** Pinki Alhyan, Anju Aggarwal, Neelam Chhillar, Sangeeta Sharma, Manish Narang, Rajeev Kumar Malhotra

**Affiliations:** 1 Pediatrics, University College of Medical Sciences, Delhi, IND; 2 Pediatrics, University College of Medical Science, New Delhi, IND; 3 Neurochemistry, Insititute of Human Behaviour and Appiled Sciences, New Delhi, IND; 4 Neuropsychopharmacology, Institute of Human Behaviour and Allied Sciences, New Delhi, IND; 5 Pediatrics, University College of Medical Sciences, New Delhi, IND; 6 Statistics, All India Institute of Medical Sciences, New Delhi, IND

**Keywords:** children, epilepsy, valproate, magnesium level, thyroid function

## Abstract

Background: Antiseizure drug valproate alters thyroid functions. Magnesium is implicated in the pathogenesis of epilepsy and it may affect the efficacy of valproate and thyroid functions.

Objective: To study the effect of six months of valproate monotherapy on thyroid functions and serum magnesium levels. To study the association among these levels and the effects of clinicodemographic profile.

Materials and method: Children aged three to 12 years presenting with newly diagnosed epilepsy were enrolled. A venous blood sample was collected for estimation of thyroid function test (TFT), magnesium, and valproate levels at onset and after six months of valproate monotherapy. Valproate levels and TFT were analyzed by chemiluminescence and magnesium by colorimetric method.

Results: Thyroid stimulating hormone (TSH) increased significantly from 2.14±1.64 µIU/ml at enrollment to 3.64±2.15 µIU/ml at six months (p<0.001), free thyroxine (FT4) decreased significantly (p<0.001). Serum magnesium (Mg) decreased from 2.30±0.29 mg/dl to 1.94±0.28 mg/dl (p<0.001). At six months, eight out of 45 (17.77%) participants had significantly increased mean TSH levels (p=0.008). Serum valproate levels were not associated significantly with TFT and Mg (p<0.05). There was no effect of age, sex, or repeat seizures on the measured parameters.

Conclusion: The TFT and Mg levels are altered by six months of valproate monotherapy in children with epilepsy. Hence we suggest monitoring and supplementation if required.

## Introduction

Epilepsy is a common neurological disorder. The incidence of epilepsy ranges from 47 to 187 per lakh population [1]. The incidence is more in underdeveloped countries and rural areas. The majority of cases start in childhood [1]. Sodium valproate is known for its broad-spectrum antiseizure activity, gastrointestinal tolerance, less sedative action, and no effect on cognition. It is a commonly used antiseizure medication [2]. Sodium valproate therapy is associated with thyroid dysfunction [3]. Various studies have been conducted to study the effect of the drug on the thyroid function test (TFT). The studies conducted by Verotti et al. and Dhodi et al. demonstrated no change in thyroid function [4,5]. Other studies by Malwade et al., Aggarwal et al., and Cansu et al. revealed a significant increase in thyroid-stimulating hormone (TSH) levels in children receiving valproate [3,6,7].

Magnesium (Mg) has been shown to affect the efficacy of sodium valproate in a mouse model [8]. Magnesium has been implicated in epilepsy by acting as a non-competitive antagonist of the N-methyl-D-aspartate (NMDA) receptor, which modulates neurotransmission by blocking voltage-dependent NMDA receptors. In vitro reduction of extracellular Mg level lowers the threshold level of excitatory amino acids necessary for activation of NMDA receptor and induces spontaneous epileptiform activity [8]. Magnesium depletion enhances oxidative stress [9]. Children on antiseizure drugs may show altered serum Mg levels [10].

The studies evaluating TFT in children on sodium valproate monotherapy have shown varying results with only a few being longitudinal studies. Hence, this study was planned to evaluate the effect of sodium valproate monotherapy on TFT and serum Mg levels in children aged three to 12 years with newly diagnosed epilepsy.

## Materials and methods

This longitudinal study was conducted at a tertiary care hospital in India from November 2018 to April 2020. Ethical clearance was taken from the Institutional Ethical Committee of the University College of Medical Sciences in New Delhi, India (approval no. IEC-HR/2018/36/113). Children were enrolled after obtaining written informed consent from their parents/legal guardians. The sample size was calculated by using a previous study by Malwade et al. [3]. The mean and standard deviation (SD) of TSH at the start of the study and six months after was 3.21±1.80 µIU/ml and 4.13±2.03 µIU/ml using G power 3.1.9.2 software (Universitat Kiel, Germany). A sample size of 37 was calculated for the power of 80% and an alpha error of 5%. It would measure an effect size of 47%. However, considering the loss of follow-up, we had to enroll 50 patients.

Children aged three to 12 years with newly diagnosed epilepsy who were to receive valproate therapy were enrolled. Those with a thyroid disorder, receiving other antiseizure drugs for 15 days or more, congenital anomalies, developmental delay, neurological diseases, chronic liver disease, and chronic renal failure were excluded from the study. Baseline demographic and clinical data were recorded. Neuroimaging and EEG were carried out. Hemograms, liver function tests, kidney function tests, electrolytes, Mantoux tests, and chest X-rays were carried out as per requirement. Valproate was administered in a loading dose of 20 mg//kg; starting maintenance dose was 10 mg/kg/day and increased to 20mg kg/day over two weeks. Children diagnosed with neurocysticercosis and tuberculoma as the cause of epilepsy received treatment (albendazole and steroids) as per hospital protocol.

Blood samples were taken on day 1 and six months after initiating therapy to estimate valproate levels. The sixth-month sample was collected in the morning before the morning valproate dose. A 3 ml blood sample was collected in a plain vial under aseptic precautions. Free triiodothyronine (FT3), free thyroxine (FT4), and TSH were measured by electrochemiluminescence immunoassay with Roche Elecsys 2010 Rack Immunology Analyzer (Roche Diagnostics Corp., Indianapolis, IN, USA). Serum Mg was measured by the Diatron Pictus Analyzer (Diatron, Budapest, Hungary) using the Xylidyl blue-I chromogenic method. The analytical sensitivity of FT3 (normal range: 2.0-4.4 pg/ml), FT4 (normal range: 0.93-1.7 ng/dl), TSH (normal range: 0.27-4.2 µIU/ml), and serum Mg (normal range: 1.6-2.7 mg/dl) was 1.18 pg/ml, 0.039 ng/dl, 0.013 µIU/ml, and 0.10 mg/dl, respectively. Children with altered thyroid function underwent an ultrasound of the thyroid and anti-thyroid peroxidase antibodies. Serum valproate level was estimated at day 1 and at six months of therapy using the CEDIATM Valproic Acid II Assay (Thermo Fisher Scientific, Waltham, MA, USA).

Data were analyzed using SPSS version 21 (IBM Corp., Armonk, NY, USA). The mean values of TSH, FT3, FT4, Mg, and valproate at enrolment and at six months were compared by paired t-test. The association between these levels was estimated by Pearson’s coefficient. The effect of the clinicodemographic profile on these variables was analyzed using chi-square (Fischer exact) for categorical variables and t-test for quantitative variables. A p-value of <0.05 was taken as significant.

## Results

A total of 50 children with newly diagnosed epilepsy between the age of three and 12 years were enrolled. Out of the 50 children, 45 were followed up till six months, and five were lost to follow-up. Data of these 45 participants were analyzed. The mean age was 7.94±2.39 years. Of these participants, 12 were (26.7%) aged between two and six years, 16 (35.6%) were aged between older than six and nine years, and 17 (37.6%) were aged between older than nine and 12 years. There were 26 males and nine females.

Table 1 shows the comparison of levels at the time of enrollment and at six months after therapy. Thyroid stimulating hormone increased significantly from 2.14±1.64 µIU/ml at enrollment to 3.64±2.15 µIU/ml at six months (p<0.001), FT3 increased significantly (p=0.021) and FT4 decreased significantly (p<0.001) at six months. Magnesium levels decreased significantly at six months (p<0.001).

**Table 1:** Thyroid function test and Mg levels at the time of enrolment and six months after therapy FT3: Free triiodothyronine, FT4: Free thyroxine, TSH: Thyroid stimulating hormone, Mg: Magnesium

Parameters	Mean±SD		Mean difference (6 months minus baseline) (95% CI)	p-value*
	At enrollment	At 6 months		
FT3 (pg/ml)	3.57± 0.72	3.90±0.63	0.32(0.05 to 0.60)	0.021
FT4 (ng/dl)	1.45±0.31	1.19±0.21	-0.27(-0.37 to -0.17)	<0.001
TSH (µIU/ml)	2.14±1.64	3.64±2.15	1.50(0.84 to 2.16)	<0.001
Mg (mg/dl)	2.30±0.29	1.94±0.28	-0.36(-0.24 to -0.06)	<0.001

Table 2 represents the number of participants with normal and abnormal levels of TFT, serum Mg, and serum valproate.

**Table 2:** Number of participants with normal/abnormal TFT, serum Mg, and serum valproate FT3: Free triiodothyronine, FT4: Free thyroxine, TSH: Thyroid stimulating hormone, Mg: Magnesium, TFT: Thyroid function test

Parameters	00	01*	10*	11	p-value
FT3	31	6	6	2	1.000
FT4	32	4	8	1	0.388
TSH	34	8	0	3	*0.008
Mg	41	2	2	0	1.000
Valproate	19	8	15	3	0.210

Comparing the levels which were normal at enrollment and abnormal at six months (01*) with levels that were abnormal at enrollment and normal at six months (10*) shows that there was a significant change in TSH levels at six months from the baseline tested using the Mc Nemar test (p=0.008).

The mean dose of valproate was 20.78±2.37 mg/kg/day, resulting in a mean serum valproate level of 72.64±25.77 µIU/ml at enrollment and 65.38±20.6 µIU/ml (reference range 50-100 mg/l) at six months. At baseline, 40% of patients were out of range (10 less than 50 mg/l and eight over 100 mg/l) and 24.4% of patients were out of range at six months (11 were less than 50 mg/l). Table 3 shows that Mg levels were not significantly associated with thyroid functions and valproate levels(p>0.05).

**Table 3:** The association of serum Mg level with TFT and serum valproate level at the time of enrolment and six months after therapy A: At the time of enrolment, B: Six months after therapy, FT3: Free triiodothyronine, FT4: Free thyroxine, TSH: Thyroid stimulating hormone, Mg: Magnesium, TFT: Thyroid function test

	FT3 A – r value*( p-value)	FT4 A – r value* (p-value)	TSH A – r value* (p-value)	Valproate A – r value* (p-value)
Magnesium A	-0.23 (0.12)	0.08 (0.60)	-0.18 (0.24)	-0.13 (0.41)
	FT3 B – r value* (p-value)	FT4 B – r value* (p-value)	TSH B – r value* (p-value)	Valproate B – r value* (p-value)
Magnesium B	-0.02 (0.99)	0.06 (0.67)	-0.05 (0.72)	0.08 (0.58)

Neuroimaging was normal in 37 participants, tuberculoma was seen in three, and neurocysticercosis in five participants. Hematological and biochemical parameters were within normal limits in all participants. Only five participants had repeat episodes of seizures in whom, after increasing the dose of valproate by 5 mg/kg, seizures were controlled. Thyroid function test and serum Mg levels were not affected by weight for age and height for age (p>0.05). But FT3 at enrollment was significantly associated with weight for height or BMI (p=0.044). In our study, TFT and serum Mg levels were not affected by age and gender (p>0.05).

## Discussion

The present study revealed a statistically significant increase in TSH levels on valproate monotherapy of six months duration (p< 0.001). A similar increase was demonstrated in previous studies by Malwade et al. and Aggarwal et al. [3,6]. However, a study done by Verotti et al. [4] did not show a significant increase in TSH levels. Measurement of serum TSH levels is considered the most reliable evaluation of the total thyroidal state of patients taking antiseizure drugs. Subclinical hypothyroidism can have clinical implications in cognition and neurobehavioural features [11,12]. This study revealed subclinical hypothyroidism (TSH >5 µIU/ml but less than 10 µIU/ml) in eight out of 45 (17.7%) participants. The incidence of subclinical hypothyroidism was similar to the study by Malwade et al. who demonstrated subclinical hypothyroidism in 21.8% [3]. Subclinical hypothyroidism was studied by Kim et al. [13]. Vainionpaa et al. demonstrated a reversal of raised TSH levels after discontinuation of the medication [14]. There was no association between serum valproate level and subclinical hypothyroidism, as in previous studies [6,15]. Though Kim et al. demonstrated an association between valproate levels and subclinical hypothyroidism [13].

The current understanding of the pathogenesis of subclinical hypothyroidism and valproate monotherapy is unclear. A hypothesis suggests that valproate increases TSH due to gamma-aminobutyric acid (GABA) stimulatory properties (GABA inhibits the release of somatostatin which inhibits TSH secretion) and it causes a decrease in the concentration of serum T4-binding globulin, displacement of thyroxine from protein binding sites, and decreased T4-deiodinase activity [15]. Another mechanism postulated for subclinical hypothyroidism is that valproate leads to zinc and selenium deficiency, and these elements are involved in important enzymes in thyroid hormone synthesis [6].

Magnesium as an enzyme cofactor, plays a critical role in mitochondrial oxidative phosphorylation and adenosine triphosphate (ATP) synthesis. According to a hypothesis, its deficiency can lead to decreased iodine uptake by thyroid cells and a subsequent drop in thyroid hormone synthesis, thereby causing the secretion of TSH (because iodine uptake is achieved by sodium iodide co-transporter that requires mitochondrial energy supply) [16]. Another mechanism is that a decrease in magnesium levels influences the action of thyrotrophic hormone on the thyroid gland through the formation of cyclic adenosine monophosphate (AMP) involved in the action of adenylyl cyclases and stimulates cyclic 3, 5 nucleotide phosphodiesterase. It has been suggested that Mg levels decreased in hypothyroidism due to increased fractional excretion of magnesium [17].

Our study was carried out on a uniform set of pediatric participants who presented with generalized tonic-clonic epilepsy requiring valproate therapy. Hence there was no diagnostic bias at enrollment. Then, the levels of the same participant were compared with levels at six months, hence results were not likely to be affected by genetics, environment, or diet. Steroids were administered for the initial few days in eight out of 45 participants which is unlikely to influence thyroid functions at six months. Whether the change in TFT was due to valproate therapy or due to epilepsy is difficult to establish as a group of children with epilepsy who are not treated would be unethical. It is observed in the literature that at times TFT reverts back to normal after discontinuation of valproate therapy [7,14]. Hence the need for long-term studies

## Conclusions

This study revealed that Mg levels decreased significantly at six months of valproate therapy. Levels of FT4 decreased and TSH increased significantly at six months of valproate therapy, 17.7% of the participants developed subclinical hypothyroidism. Periodic monitoring and appropriate supplementation of Mg and thyroxine are suggested.
